# Long-Term Efficacy of the Workshop Vs. Online SUCCEAT (Supporting Carers of Children and Adolescents with Eating Disorders) Intervention for Parents: A Quasi-Randomised Feasibility Trial

**DOI:** 10.3390/jcm9061912

**Published:** 2020-06-18

**Authors:** Stefanie Truttmann, Julia Philipp, Michael Zeiler, Claudia Franta, Tanja Wittek, Elisabeth Merl, Gabriele Schöfbeck, Doris Koubek, Clarissa Laczkovics, Hartmut Imgart, Annika Zanko, Ellen Auer-Welsbach, Janet Treasure, Andreas F. K. Karwautz, Gudrun Wagner

**Affiliations:** 1 Eating Disorders Unit, Department of Child and Adolescent Psychiatry, Medical University of Vienna, 1090 Vienna, Austria; stefanie.truttmann@meduniwien.ac.at (S.T.); julia.philipp@meduniwien.ac.at (J.P.); michael.zeiler@meduniwien.ac.at (M.Z.); claudiaparfuss@gmx.at (C.F.); tanja.wittek@meduniwien.ac.at (T.W.); elisabeth.merl@outlook.com (E.M.); gabriele.schoefbeck@meduniwien.ac.at (G.S.); praxis@kiju.co.at (D.K.); clarissa.laczkovics@meduniwien.ac.at (C.L.); andreas.karwautz@meduniwien.ac.at (A.F.K.K.); 2 Parkland Clinic, Clinic for Psychosomatic Medicine and Psychotherapy, 34537 Bad Wildungen, Germany; hartmut.imgart@parkland-klinik.de (H.I.); annika.zanko@parkland-klinik.de (A.Z.); 3 Department for Neurology and child and adolescents Psychiatry, 9020 Klagenfurt am Wörthersee, Austria; Ellen.Auer-Welsbach@kabeg.at; 4 Section of Eating Disorders, Department of Psychological Medicine, Institute of Psychiatry, Psychology & Neuroscience, King’s College London, London WC2R 2LS, UK; janet.treasure@kcl.ac.uk

**Keywords:** anorexia nervosa, children and adolescents, parents, carers, intervention, workshop, online intervention

## Abstract

Interventions for main carers of adult patients with anorexia nervosa (AN) can reduce the caregiving burden and increase caregiver skills. However, the effectiveness and feasibility for carers of adolescent patients, the optimal form of the intervention and long-term outcomes are largely unknown. We evaluated the efficacy and feasibility of the “Supporting Carers of Children and Adolescents with Eating Disorders in Austria” (SUCCEAT) workshop vs. online intervention. Main caregivers (parents) of adolescent patients with AN were randomly allocated to a workshop (*n* = 50) or online version (*n* = 50). Participants were compared to a non-randomised comparison group (*n* = 49) receiving multi-family or systemic family therapy. Primary (General Health Questionnaire) and secondary outcomes were obtained at baseline, three-month and 12-month follow-up. Adherence was high for workshop and online participants (6.2 and 6.7 sessions completed out of 8). Intention-to-treat analyses revealed significant pre–post reductions in the primary outcome for the workshop (d = 0.87 (95%conficence interval (CI): 0.48; 1.26)) and online (d = 0.65 (95%CI: 0.31; 0.98)) intervention that were sustained at the 12-month follow-up. There was no significant group difference (*p* = 0.473). Parental psychopathology and burden decreased and caregiver skills increased in all groups; the improvement of caregiver skills was significantly higher in SUCCEAT participants than in the comparison group. Online interventions for parents of adolescents with AN were equally effective as workshops. The improvements remained stable over time.

## 1. Introduction

The National Institute for Health and Care Excellence (NICE) Guidelines recommend involving carers in the treatment of adolescent patients with anorexia nervosa (AN) [1]. As AN tends to develop during adolescence, parents usually function as main carers. AN is a life-threatening psychiatric disorder that is often related to a high mortality and a high chronification rate [2]. Furthermore, AN is often associated with lack of insight and low motivation to change in patients. Therefore, the illness can have a serious emotional impact on the whole family, especially the main carer, who usually is a parent. This can cause severe parental distress and lead to enormous burden and relationship problems within the whole family [3,4,5,6].

In the cognitive interpersonal model of maintaining factors for eating disorders (ED), Treasure and Schmidt [3] exemplify how interpersonal relationships between caregivers and patients play an important role in the recovery of patients with AN. They illustrate how unhelpful parental reactions, overflowing parental emotions of any kind and dysfunctional parental communication styles may maintain AN symptoms and therefore lead to a vicious circle of dispute, avoidance and misunderstanding, which might worsen the parents’ and patients’ outcome as a result: It may not only hinder the recovery of the patients but might also lead to clinically relevant anxiety and depression in caregivers themselves [7,8]. Therefore, it is essential to teach main carers, mostly parents, specific skills and communication styles to help them handle difficult situations, in order to break the vicious circle. As confirmed by a meta-analysis of interventions for caregivers of mainly adult patients suffering from AN [9] that are based on the cognitive interpersonal model of maintaining factors for eating disorders (ED) [3,10,11], they can reduce a carer’s burden and improve caregiver skills.

Usually, these interventions are delivered as workshop [12,13] or self-help programmes [14,15,16]. Only a few studies examined online versions, but mainly for carers of adult patients [17,18,19]. Online interventions have the advantages of flexibility in time and place and may facilitate professional support when face-to-face appointments are not possible or specialists are not available in remote areas. Moreover, participants may experience less anxiety, shame or stigmatization during online counselling [11,20,21]. So far, there is not enough evidence whether online interventions are as effective as face-to-face interventions in the field of ED, especially in terms of carers of adolescent patients [22]. To investigate the efficacy of online interventions seems even more essential regarding the current coronavirus disease 2019 (COVID-19) pandemic, when face-to-face contact should be restricted as much as possible. Although there are still some barriers, such as online privacy and lack of sufficiently free access to mental health service programmes, they offer a lot of opportunities when it comes to the need of high safety [23]. Moreover, online programmes have the potential to provide help for families that may otherwise have difficulties accessing clinical help. However, a recent systematic review [22] reported only eleven studies examining online carer interventions on carer mental health, with two studies focusing on AN [17,18].

Many studies have a short follow-up (FU) period, usually between three [14,18,19] and six months [17]. Long-term outcome was rarely considered in previous research.

The majority of these studies mainly included carers of adult patients [12,14,24]. Evidence for the effectiveness for carers of adolescents is scarce, with only one study focusing solely on adolescents [15]. Comparisons between workshop and online versions are completely missing for carers of adolescent patients. To evaluate interventions for carers of adolescents is essential, as AN tends to start in adolescence and early interventions are known to positively affect its course [1,25]. Many carers of adult patients express a wish to have had early access to such an intervention [13].

Altogether, the effectiveness and feasibility of caregiver interventions for carers of adolescent patients and whether an online version is equally effective and feasible as a face-to-face format is largely unknown. The aim of this study was to compare the feasibility and explore the potential long-term efficacy of “Supporting Carers of Children and Adolescents with Eating Disorders in Austria” (SUCCEAT) programme delivered as a workshop or online programme for carers (usually parents) of adolescent patients. The objective of SUCCEAT is to decrease carers’ burden by accrediting them with skills and knowledge, in order to increase their ability to better support their affected children and adolescents [26]. We hypothesise that parents participating in SUCCEAT show a statistically significant reduction of distress, burden and psychiatric symptoms at the post-intervention and at 12-month FU. Furthermore, we aimed to figure out whether the intervention effects are different when delivering the programme via workshops or online modules.

## 2. Methods

### 2.1. Study Design

Reporting to this study adheres to the CONSORT (Consolidated Standards of Reporting Trials) guidelines (see online Supplementary Materials “ESM1. Consort checklist”). This is a two-arm parallel group quasi-randomised feasibility trial that was conducted between November 2014 and April 2019. Carers of children and adolescents between 10 and 19 years suffering from AN or atypical AN were eligible to participate in the study. In this study, all main caregivers were parents living in the same household as the patients they cared for. The inclusion criterion for caregivers was German fluency. The inclusion criterion for patients was receipt of treatment as usual, according to NICE guidelines [1]. Participants were excluded if caregivers suffered from a severe mental illness or patients suffered from severe comorbidities at baseline (e.g., psychosis) and if carers lacked Internet access. The SUCCEAT group (workshop/online) was compared to a non-randomised comparison group of carers receiving other forms of family support. Details of the study protocol are published elsewhere [26]. The study was registered at ClinicalTrials.gov (Identifier: NCT02480907).

### 2.2. Recruitment and Randomisation

The study protocol and the informed consent forms were approved by the Ethical Commission of the Medical University of Vienna (#1840/2013). For each patient, one parent served as main carer and completed the questionnaires. SUCCEAT participants were recruited at the Medical University of Vienna (Department of Child and Adolescent Psychiatry). Patients underwent psychiatric and psychological assessments. If eligible, parents were informed about the study and invited to participate. After providing written informed consent, carers completed the baseline assessments and were allocated to one of the intervention-arms. The practicalities of recruitment (limited number of study participants to be enrolled in a reasonable period of time) meant that we were not able to implement a technically exact randomisation procedure (blockwise in blocks of eight participants) as originally planned and described in the study protocol [26]. Rather, we fixed the start dates of the workshop and online groups in advance and alternately assigned the first block of enrolled participants to the workshop, the next block to the online intervention and so forth. This procedure corresponds to the definition of quasi-randomisation as reported in the Cochrane Handbook for Systematic Reviews (Box 13.4a) [27]. The block sizes slightly varied depending on the number of incoming participants prior to the start of a group (median size = 7). We assumed that the time of enrollment of participants is not systematically related to any participants’ characteristics. We tested whether participants allocated to the workshop and online group differed regarding sociodemographic characteristics, clinical characteristics of patients and baseline scores of the outcome questionnaires and found that solely patients of parents assigned to the online group had slightly longer ED duration compared to the workshop group, while there were no other statistically significant differences (see Results section for details). The participants were not informed about the group allocation until baseline assessments were completed. Caregivers who did not want to participate in the intervention to which they were assigned (*n* = 2 in online group) were excluded. Researchers involved in this study were not blinded to the group allocation.

The comparison group was recruited from the Department for Neurology and Child and Adolescent Psychiatry (Klagenfurt, Austria) and the Parkland Clinic, Clinic for Psychosomatic Medicine and Psychotherapy (Bad Wildungen, Germany); two clinics specialised in ED treatment. The same procedure for checking eligibility and obtaining informed consent was applied.

### 2.3. Interventions

SUCCEAT was delivered either via workshop or online. Both intervention arms were designed equally, including 8 weekly modules. The SUCCEAT intervention teaches carers about common emotional and behavioural interactions that may occur when living with a person suffering from anorexia nervosa, such as dysfunctional communication and misattributions. Carers are taught skills to counter these reactions, such as Motivational Interviewing, problem solving, resilience and difficult-behaviour management [5,8]. Caregivers had either face-to-face contact (in the workshops) or had the opportunity to exchange thoughts via an online forum. Two health professionals coached both the workshop and the online intervention. One was a child and adolescent psychiatrist, and the other one was a psychologist and medical doctor in child and adolescent psychiatry training. Both worked in the field of ED treatment and research for several years and were trained in Motivational Interviewing.

#### 2.3.1. Workshop Group

The two healthcare professionals (subsequently referred to as “coaches”) delivered these modules in eight weekly sessions. Caregivers received handouts in each session, as well as a comprehensive manual based on a book [28], including detailed information, and a DVD [29] with case examples of caregiver–patient interactions.

#### 2.3.2. Online Group

Caregivers were invited to a face-to-face welcome meeting, where they got to know the coaches and each other. Participants got access to the online programme, the handouts and the manual and were asked to complete one module weekly. They also received the DVD. Once a week, they received written feedback regarding their progress and responses to questions by one of the coaches.

#### 2.3.3. Comparison Group

Comparison-group participants received either four double sessions of systemic family therapy (Klagenfurt, Austria) or multi-family therapy (Bad Wildungen, Germany). Multi-family therapy, based on the Maudsley Model of multi-family therapy for AN, is conducted in a two-day face-to-face workshop delivered by a therapeutic team, including up to 10 families [30].

### 2.4. Assessments

The parents completed self-report questionnaires at baseline (T0), after the intervention (3-month FU, T1) and at 12-month FU (T2). They rated their own psychopathology and caregiving skills. The evaluation included the following assessments:

The 12-items version of the General Health Questionnaire (GHQ) assesses the level of parental psychological distress [31,32]. Items are rated on a four-point scale (0–3) and are converted to a dichotomous rating (“0” for the two lowest ratings; “1” for the two highest ratings) and summed up to a total score ranging from 0 to 12 (higher scores indicating higher levels of psychological distress).

The Eating Disorder Symptom Impact Scale (EDSIS) assesses specific parental caregiving difficulties for families of people with EDs [33]. A total of 24 items rated on a five-point scale are aggregated to a total score and four sub-scores indicating difficulties in specific areas. Higher scores indicate more difficulties. Subscales comprise difficulties related to “nutrition” (e.g., difficulties preparing meals or arguments during mealtimes), “dysregulated behaviour” (e.g., temper outbursts or lying), feelings of “guilt” (e.g., feelings of having done something wrong) and “social isolation” (e.g., losing friends).

The Symptom Checklist (SCL-90-Revised) [34] consists of 90 items rated on a 5-point scale assessing a broad range of psychopathological symptoms which parents might develop over time while caring for a child with AN (e.g., somatization, obsessive–compulsive behaviour, etc.). For the purpose of this study, only the global severity index (sum score of all items divided by 90; score range: 0–4) was calculated with higher scores, indicating higher levels of psychopathology.

The Beck Depression Inventory (BDI-II) [35,36] comprises 21 items assessing symptoms of parental depression, rated on a four-point scale. Items are aggregated to a total score, with higher scores indicating higher levels of depression.

The State and Trait Anxiety Inventory (STAI) [37], consisting of 40 items rated on a four-point scale, measures two kinds of anxiety. “State-anxiety” is characterised as inner tension and concerns toward future events in carers that vary across time and situations; “trait anxiety” means the tendency of experiencing fear in general. Higher scores indicate higher anxiety.

The Caregiver Skills (CASK) scale [38] measures skills in parents caring for ED patients and included 27 items rated on a visual analogue scale (0–100) which are summed up to the following subscales: “Bigger Picture” (implementing bigger-picture thinking), “Self-Care” (taking care of yourself), “Biting Tongue” (avoiding repetitive, nagging arguments), “Insight and Acceptance” (accepting and managing negative emotions), “Emotional Intelligence” (ability to discuss and manage feelings) and “Frustration Tolerance” (ability to be firm, calm and understanding). Item ratings are aggregated to one total scale and six subscales. Mean scores were calculated for this study, with higher scores indicating higher skill levels.

Furthermore, sociodemographic characteristics of the carers, including gender, age, highest educational degree, marital status and ethnicity, as well as patient characteristics, including gender, age, AN subtype, illness duration, type of current treatment and Body-Mass-Index (BMI), were obtained. Any adverse events reported by the caregivers were recorded during the course of the trial.

### 2.5. Statistical Analysis

Data analyses were performed with IBM SPSS Statistics 25.0 and *R*. Differences in sociodemographic and ED-related characteristics of carers and patients between the groups were analysed with *t*-tests and *χ*^2^-tests. Adherence to the SUCCEAT intervention was explored by calculating the mean number of completed workshop/online sessions and the percentage of carers who have completed more than four sessions. Furthermore, completion of the programme was defined as having completed the last session; full completion was defined as having completed the last session and at least two of the three assessments. Differences in adherence measures between the workshop and online group were explored by using *t*- and *χ*^2^-tests. Treatment effects of the SUCCEAT interventions were analysed by using a series of general linear mixed models, including outcome scores from the baseline, 3-month and 12-month FU assessments as within-factor and study arm as between-factor. While the main effect of time represents the change of scores across both SUCCEAT study arms, the time *x* group interaction represents differences in changes between the study arms. Bonferroni-adjusted *p*-values account for multiple testing when results on questionnaire subscales are presented. Baseline to 3- and 12-month FUs’ effect sizes (Cohen’s d, including 95% confidence intervals (CI)) were calculated separately by study arm.

Intention-to-treat (ITT) was chosen as the primary analytic strategy. Therefore, participants were analysed within the study arm they were randomised to, regardless of adherence. Missing outcome data were replaced by using the expectation-maximization (EM) method, which is based on maximum-likelihood estimates. Missing data were replaced separately for each outcome variable by using data from other assessment time points (baseline, 3- and 12-month FUs) and study arm as predictors (assuming normal distribution and using 25 iterations). Missing FU data were not significantly related to baseline scores or sociodemographic characteristics. Additionally, a completer analysis was conducted, including only data from carers who completed more than four workshop/online sessions and assessments from all time points. Missing data were not replaced. Thus, this analysis refers to the subgroup of carers highly adherent to both the intervention and assessments.

The same analytic strategy was applied for exploring differences between the SUCCEAT intervention group (including aggregated data from the workshop and online study arm) and the comparison group. As there was no difference in effects between comparison participants recruited from Bad Wildungen and Klagenfurt, data obtained from both sites were analysed in tandem.

## 3. Results

### 3.1. Participants

**Figure 1:**
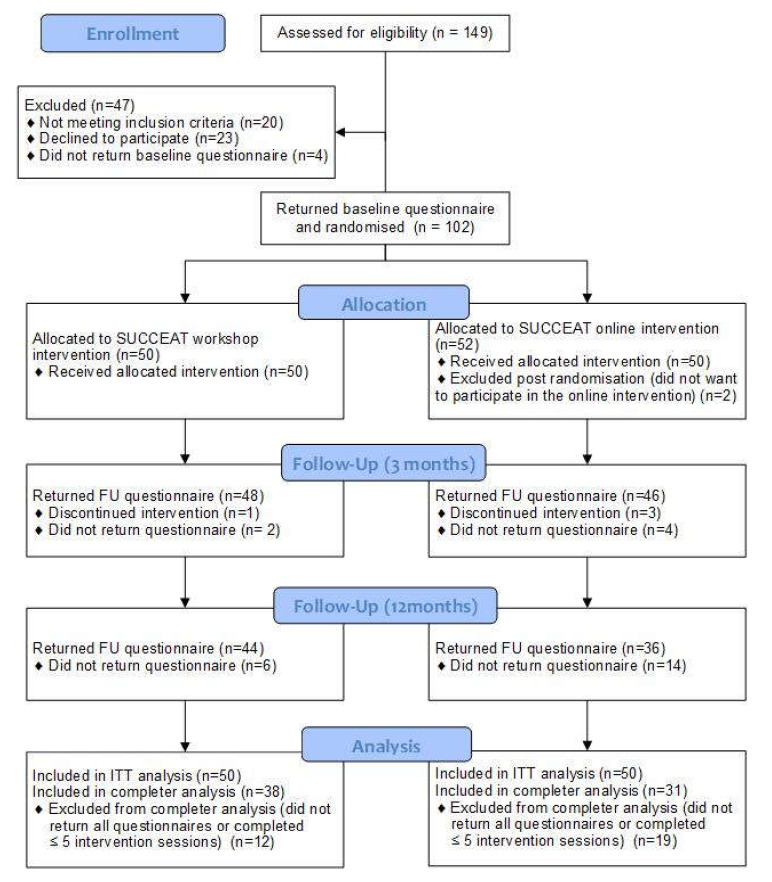
Consort flow diagram. SUCCEAT: Supporting Carers of Children and Adolescents with Eating Disorders in Austria; ITT: Intention-to-treat; FU: Follow-up.

SUCCEAT participants were 102 carers (86% females, mean age: 47.2 years) who were randomised to either the workshop (*n* = 50) or the online (*n* = 52) intervention group. Two participants were excluded post-randomisation because they were not willing to participate in the group they were randomised to, and thus they were not considered in the subsequent analyses. The participants’ flow is shown in the consort diagram (Figure 1). Baseline demographic and clinical characteristics of carers and patients are shown in Table 1. No statistically significant differences between participants of the two groups were observed. The only exception was that the AN duration was slightly longer in patients of carers assigned to the online group compared to the workshop group (*p* = 0.041). Furthermore, there were no statistically significant differences between the groups in any outcome variable (GHQ, EDSIS, BDI, STAI, SCL and CASK scores) at baseline, indicating that the quasi-randomisation approach was successful in building two comparable groups. One carer in the workshop group and three carers in the online group discontinued the interventions. Non-completion of the three-month and 12-month FU assessments was 4% and 12% in the workshop group, respectively, and 8% and 28% in the online group. Finally, data from 38 participants of the workshop and 31 participants of the online group were included in the completer analysis.

Participants of the comparison group were 49 carers (38 from Bad Wildungen, Germany; 11 from Klagenfurt, Austria) who were recruited over the same time period as the SUCCEAT group and provided consent and completed baseline assessments. The recruitment rate (caregivers who participated in the trial of those approached) was 71%. Statistically significant baseline differences between participants of the SUCCEAT and comparison group were found regarding parental educational degree, AN duration and type of treatment in patients. Participants of the comparison group were significantly less educated (*p* = 0.015) and cared for patients with a longer duration of AN (*p* < 0.001) treated more often in inpatient settings (*p* < 0.001). In the comparison group, non-completion of the three- and 12-month FU assessments was 33% and 55%, respectively, leaving 22 included in the completer analysis.

### 3.2. Adherence

On average, SUCCEAT workshop participants completed 6.16 (SD: 1.81), and online participants 6.70 (SD 2.19), out of eight sessions, which was not significantly different between the groups (*t*(98) = 1.344, *p* = 0.182). Completion and full completion rates of the intervention were 72% (workshop) and 70% (online) (Chi^2^ (1) = 0.049; *p* = 0.826). Furthermore, the percentage of participants completing more than half of sessions (workshop: 84%, online: 78%) did not differ between the groups (Chi^2^ (1) = 0.585, *p* = 0.444). Regarding treatment engagement, 51.1% of workshop and 34.8% of online participants read at least 50% of the manual, and 37.2% of workshop and 43.5% of online participants watched at least 50% of the DVD.

### 3.3. Primary Outcome

Within groups, the ITT analysis (Table 2) revealed a statistically significant reduction in the carers’ burden, as indicated by the GHQ total score across both SUCCEAT interventions (F = 36.252, *p* < 0.001). Baseline-to-post effects sizes were d = 0.87 (95%CI: 0.48; 1.26) in the workshop and d = 0.65 (95%CI: 0.31; 0.98) in the online group. Baseline-to-12-months FU effect sizes were d = 0.81 and 0.98, respectively. Results from the completer analysis (Table S1 in online “ESM2. Supplementary Tables S1–S4”) parallel the results of the ITT analysis with a slightly higher baseline-to-12-months FU effect in the online group).

Between-groups changes in GHQ scores across the time points were not significantly different between the SUCCEAT groups (F = 0.752, *p* = 0.473), thus indicating no difference in the treatment effect for the workshop and online group.

### 3.4. Secondary Outcomes

Results of the general linear mixed model analyses calculated on an ITT basis are shown in Table 2 and Table 3.

ED-related burden: The ITT analysis revealed statistically significant reductions in caregivers’ burden related to AN symptomatology across SUCCEAT interventions, which was persistent to 12-month FU. Effect sizes were highest for the EDSIS nutrition subscale (large effects), whereby effect sizes for the other subscales were in the low-to-medium range. There was no statistically significant difference between the workshop and online group for the EDSIS total score or subscales.

Psychopathology: Carers’ general psychopathology, as measured with the SCL-90 R, was significantly reduced over time, across both SUCCEAT intervention-arms, while there was no statistically significant time × group interaction effect in the ITT analysis. Significant long-term reductions in symptom levels were also observed for depression and anxiety scores across both SUCCEAT groups.

Caregiver skills: From baseline to three- and 12-month FUs, caregiver skills as measured with the CASK significantly increased across both SUCCEAT intervention-arms, while there was no statistically significant time × group interaction in the total and any of CASK subscales (Table 3). Effect sizes were medium to high.

Results of the completer analyses for secondary outcome measures (Tables S1 and S2 in “ESM2. Supplementary Tables S1–S4”) were largely congruent with results of the ITT analyses, but with two exceptions: For the SCL-90-R score, the completer analysis revealed higher reductions in the online compared to the workshop group (F = 4.467, *p* = 0.014). Furthermore, the statistically significant interaction effect for the “Emotional Intelligence” subscale of the CASK observed in the ITT analysis was not found in the completer analysis.

Relation to comparison group: As indicated by the statistically significant main effects of the group in the ITT analysis (Table S3 in “ESM2. Supplementary Tables S1–S4”), carers of the comparison group receiving either multi-family therapy or systemic family therapy generally scored higher across all time points in all burden and psychopathology measures than participants of the SUCCEAT groups. Baseline to three-month FU effect sizes were slightly higher in SUCCEAT participants, as compared to participants from the comparison group. However, the time × group interaction did not reach statistical significance for any outcome measure, except for the CASK total score, where caregiver skills increased significantly more in SUCCEAT participants, as compared to participants of the comparison group (F = 5.570, *p* = 0.004). Considering data from completers only (Table S4 in “ESM2. Supplementary Tables S1–S4”), only statistically significant main effects of time were observed.

### 3.5. Adverse Events

No harms were reported by any caregiver, and no patient died during the trial.

## 4. Discussion

We evaluated the efficacy of the SUCCEAT programme for carers of adolescents with AN and focused on a potential difference between using a workshop or online format for delivering the content. Within both groups, the primary outcome and all secondary outcomes improved. Caregivers reported a statistically significant reduction in distress, burden, anxiety and depression, as well as a statistically significant increase in caregiver skills. These improvements were maintained to the one-year FU. Our study, therefore, confirms that interventions for caregivers of adolescents patients with AN based on the cognitive interpersonal model [3,10,11] are (1) effective for caregivers of an adolescent population and (2) independent from the type of intervention (workshop or online).

Adherence of SUCCEAT was high. Completion rates of SUCCEAT were high (70–72%) compared to a systematic review of online interventions for patients with ED and carers with a range of 18.4–95.5% [39]. Full completion was as high as completion, indicating that participants who completed the intervention also completed at least one of the FU assessments. Thus, compliance to participate in the assessments was high. However, compared to other guided interventions for caregivers of adult patients with ED based on the same theoretical framework as SUCCEAT, the adherence was similar [17,18,19,24], but higher than in other reports investigating self-help material [15,16]. “Overcoming Anorexia Online”, which was investigated in different studies [17,18,19,24], seems comparable to the concept of SUCCEAT, as it is also designed as an interactive, multi-media intervention consisting of eight modules that were presented offline or online, mostly with additional guidance that usually was provided by specialised clinicians. On the contrary, adherence of SUCCEAT was better than in studies investigating self-help with guidance from less-experienced coaches [15,16]. Even though the content was similar in all these approaches, a more diversified, structured and professionally guided presentation, as it was offered in SUCCEAT, may have positively influenced engagement. Moreover, adherence in the online intervention may have been facilitated by the welcome meeting with the coaches and other carers, as personal contact with the coaches may improve the commitment to complete the intervention. Furthermore, patients received treatment at the same facilities where the interventions were offered, enabling a conjoint case-management that may enhance the compliance in caregivers. Interestingly, the results of the ITT and completer analysis are similar, indicating that the programme effects were robust across high- and less-compliant participants.

Furthermore, a higher proportion of workshop participants read at least half of the manual, compared to the online participants. This difference may be explained by the fact that online participants already had to read all of the information at the online platform and thus may not be willing to spend even more time reading additional contents, whereas workshop participants were offered a face-to-face presentation of the material and may have been more willing to read the additional information. Otherwise, more online participants watched more than half of the DVD, as compared to the workshop participants. This may be due to the fact that communication skills could easily and concretely be demonstrated in the workshops by the coaches, but not so much online. Therefore, online participants probably had to harken back to the DVD more often. However, to what extent the additional material was used was left to the parents. Parents were regularly encouraged to gather more information on specific topics if they felt the need to, but the focus was clearly on the participation of the workshop or the online programme. However, not reading the whole manual and not watching the whole DVD might not be clinically relevant, as the topics in the additional material are fully covered within the workshops or online modules. Anyway, the engagement to read the manual and watch the DVD was better in SUCCEAT than in a previous study using self-help material [15], even though the manual and the DVD were the only materials offered in that specific study.

Carers’ distress (GHQ) was reduced significantly over the course of the SUCCEAT intervention. Furthermore, psychiatric symptoms (anxiety and depression) in caregivers decreased. ED specific burden also significantly decreased with the largest effects on the “Nutrition” subscale. This could be due to the fact, that the items of this subscale (e.g., difficulties preparing meals, tension during mealtimes, etc.) were intensively addressed in the SUCCEAT intervention. Caregivers of ED patients are known to report a lack of support especially with meal-times [40]. Therefore, this is an important topic to address, as situations around meal-times are clearly associated with high frustration and extreme emotional reactions in caregivers. Skills and communication techniques that aim to improve meal-times and associated situations were repeatedly demonstrated in the workshop and the online programme of SUCCEAT. In addition, this information was also considered in detail on the DVD, which included specific scenarios of unhelpful and helpful communication between patients and carers, especially around meal-times. Reducing the burden and improving skills in that domain may support a better outcome of the affected patient by improving confidence in caregivers to support their children and adolescents, especially in these fundamental everyday situations.

Moreover, statistically significant improvements in all domains of caregiver skills was found, which seems unique for the SUCCEAT intervention. It is assumed that stabilising the mental health of caregivers and improving their skills may positively affect patients’ outcomes as well [3,10,11,16]. Emotional regulation or positive communication may support recovery in patients with ED, and therefore, interventions that increase caregiving skills in carers, are seen as an important add-on in patients’ treatment.

In summary, the SUCCEAT intervention revealed remarkably high effect sizes in both groups, as compared to prior studies [9,15,16,19]. In SUCCEAT, highly structured material with guidance from professional coaches was used. In previous studies, carers specifically reported a lack of information from professional healthcare workers and emphasised the wish for contact with clinicians [40,41]. Naturally, participants of the workshop group could ask questions as often as they wanted and received individual feedback from the professional coaches during the workshop sessions. Likewise, participants of the online arm had the possibility to write as many messages to the coach as they wanted while working through the online sessions, even though they only received one message from the coach per week, meaning that they also had multiple options to contact the coach. Both SUCCEAT intervention arms were delivered and guided by the same two coaches trained in Motivational Interviewing and with a high level of experience in the field of adolescent EDs, which has previously found to moderate outcome [22]. Guidance from experienced clinicians may contribute to higher effect sizes [17,24].

The SUCCEAT workshop and online formats appeared to be equally effective, although the study did not have sufficient power to detect moderately sized between-group effects. There already is evidence that online interventions can support recovery from ED and prevent chronicity in patients [39,42,43] and can reduce burden and increase skills in carers of adult patients with ED [17,18,19]. This study adds to the literature that online interventions are also effective for carers of adolescent patients with ED. Online interventions are promising in terms of effectiveness and also acceptability [22]. They are of great benefit, as they are transregional, highly flexible, widely accessible at any time and delivered with minimal resources. Even coaches when responding via email can provide support with high flexibility. Online programmes have the potential to provide information for carers that may otherwise experience difficulties to access help or to contact clinicians. This is even more important in times of the current COVID-19 pandemic, when professionals’ face-to-face support is partially restricted in highly affected areas. As a result of associated uncertainty, ED symptoms, anxiety and burden within the whole family might increase [44]. Thus, as there are limited possibilities to contact clinicians face-to-face, online interventions like SUCCEAT may actually gain importance.

Caregiver skills increased more in the SUCCEAT group than in the comparison group, indicating that the main target of the SUCCEAT intervention (increasing skills on how to care for adolescents with an AN) was reached. Every content obtained in the subscales was intensively and repeatedly addressed in the SUCCEAT intervention. Whereas the topics of some subscales of the CASK were part of multi-family or systemic family therapy as well (e.g., ‘Emotional Intelligence’ or ‘Insight and Acceptance’), most seem to be approached more deeply in SUCCEAT (e.g., “Self-Care”), and some seem unique for the SUCCEAT intervention (e.g., “Bigger Picture”) [30,45]. Besides, the SUCCEAT intervention offers more time to develop and strengthen these skills. Moreover, carers may benefit from the opportunity to participate in this programme without the presence of the patients, in contrast to carers of the comparison group, where patients were included in the family treatment. However, this group difference must be interpreted with caution, considering the baseline differences between SUCCEAT and comparison group participants. Moreover, there were no further statistically significant differences between SUCCEAT and the active comparison group for the primary and other secondary outcomes measures. Regarding baseline data, the comparison group showed higher scores for burden and psychopathology measures, which may be due to longer illness duration or more severe courses.

This study has several strengths: This is the first study to compare the efficacy of a workshop- and online-based intervention for carers of adolescents suffering from AN, including long-term outcomes. A core strength of this study is the usage of the same design in both interventions, containing eight modules with the same content delivered within the same time frame. Both had access to clinical guidance (either in the workshops or via weekly online messages) and the possibility to exchange personal experiences with other carers (either in the workshops or via online forum). Furthermore, the coaches were the same in both groups. We found high fidelity to the intervention in that carer skills improved, and adherence was good.

This study also has some limitations: We included carers of AN patients only. More work is needed to investigate the effects of interventions for carers of adolescent patients with bulimia nervosa and binge-eating disorder. We could not compare the efficacy of SUCCEAT to a randomised control group. Other inpatient and outpatient psychiatric facilities that have been asked to recruit control-group participants with treatment-as-usual declined. Consequently, only two units that already implemented specialised family interventions (multi-family or systemic family therapy) in their routine ED treatment concept participated. Therefore, we could only compare the efficacy of SUCCEAT to these well-established treatments, rather than to caregivers without receiving any such intervention. Moreover, the participants of the comparison group were recruited from different facilities partially located in a different country (Germany) from the SUCCEAT participants. Although the core principles of ED treatment should be comparable across all facilities involved, slight differences in treatment approaches cannot be ruled out. Besides, a direct comparison between the groups may be further impeded by different numbers of intervention sessions and baseline differences between participants of the SUCCEAT and comparison groups. Furthermore, dropout at FU assessments was higher in the comparison group than in the SUCCEAT group. The three-months dropout might have been lower for the SUCCEAT groups because the three-month assessments were conducted shortly after the SUCCEAT intervention was finished, whereas the assessment was not temporally correlated with the end of the family intervention in the comparison group. For the differences regarding the dropout rates at the 12-month FU, we assume that the participants who already filled out two assessment batteries might also be more willing to complete the final FU assessment. These differences have to be taken into account when comparing the SUCCEAT group to the comparison group. Nevertheless, the current study provides a rough estimation of the effects of SUCCEAT compared to other well-established caregiver interventions. Further research is needed, including a randomised control group and larger group sizes in all arms, to confirm the effectiveness of SUCCEAT. Data on patient outcomes are needed to investigate whether the reduction of burden and improvement of skills in caregivers positively influences ED outcomes as well.

## 5. Conclusions

This study provides support for the efficacy of SUCCEAT, an intervention for carers of adolescents with AN, reducing carer’s burden, distress and psychopathology and improving caregiver skills with high adherence and long-lasting medium-to-high effects underpinning its clinical relevance. SUCCEAT can easily be delivered and disseminated. Both the workshop and the online version can be integrated into clinical routine care of inpatient and outpatient adolescents with AN, as an adjunct to the treatment. Guidance by trained professionals and conjoint case-management at the same facility seem important for the efficacy and adherence of the intervention.

## Figures and Tables

**Table 1:** Sociodemographic characteristics of the “Supporting Carers of Children and Adolescents with Eating Disorders in Austria” (SUCCEAT) workshop and online group participants and corresponding patients.

	SUCCEAT Workshop Intervention (N = 50)	SUCCEAT Online Intervention (N = 50)	Group Difference	
			Test Statistic (df)	*p*
Carers (SUCCEAT Participants)				
Females (N, %)	42 (84.0%)	44 (88.0%)	*χ*^2^ (1) = 0.332	0.564
Age (mean, SD)	46.64 (5.43)	47.72 (4.25)	*t*(98) = 1.108	0.271
Highest educational degree				
university degree (N, %)	30 (60.0%)	23 (46.0%)	*χ*^2^ (2) = 2.885	0.236
A level degree (N, %)	11 (22.0%)	11 (22.0%)		
<A-level degree (N, %)	9 (18.0%)	16 (32.0%)		
Marital status				
single (N, %)	0 (0.0%)	3 (6.3%)	*χ*^2^ (2) = 3.481	0.175
married or living in partnership (N, %)	42 (84.0%)	36 (75.0%)		
divorced or widowed (N, %)	8 (16.0%)	9 (18.8%)		
Ethnicity			*χ*^2^ (1) = 0.000	1.000
Caucasian (N, %)	49 (98%)	49 (98%)		
Asian (N, %)	1 (2%)	1 (2%)		
**Patients**				
Females (N, %)	45 (90.0%)	48 (96.0%)	*χ*^2^ (1) = 1.382	0.240
Age (mean, SD)	14.66 (1.91)	15.12 (1.80)	*t*(98) = 1.238	0.219
Eating disorder diagnosis				
Anorexia nervosa—restrictive type (N, %)	45 (90.0%)	45 (90.0%)	*χ*^2^ (2) = 0.000	1.000
Anorexia nervosa—binge/purging type (N, %)	4 (8.0%)	4 (8.0%)		
Atypical anorexia nervosa (N, %)	1 (2.0%)	1 (2.0%)		
Eating disorder duration in months (mean, SD)	10.41 (7.10)	16.03 (16.03)	*t*(87) = 2.076	0.041
Type of treatment				
Inpatient (N, %)	24 (48.0%)	24 (48.0%)	*χ*^2^ (1) = 0.000	1.000
Outpatients (N, %)	26 (52.0%)	26 (52.0%)		
BMI at baseline (Mean, SD)	15.53 (2.13)	16.36 (2.54)	*t*(96) = 1.743	0.084

SD: standard deviation; BMI: Body-Mass-Index.

**Table 2:** Means (SDs) and results of the mixed-effects model repeated measures ANOVA for outcomes of the SUCCEAT workshop vs. SUCCEAT online intervention regarding caregiving burden and psychopathology (intention-to-treat analysis).

	Mean (SD)			ANOVA (F, *p*)			Cohen’s d (95% CI Lower; Upper)	
	Baseline (T0)	3M FU (T1)	12M FU (T2)	Group	Time	Time × Group	T0-T1	T0-T2
GHQ Total Score ^a^								
SUCCEAT Workshop	4.41 (3.15)	1.84 (2.74)	1.94 (1.39)	0.065 (0.800)	**36.252 (<0.001)**	0.752 (0.473)	0.87 (0.48; 1.26)	0.81 (0.42; 1.21)
SUCCEAT Online	4.33 (3.76)	2.14 (2.92)	1.39 (2.18)				0.65 (0.31; 0.98)	0.98 (0.50; 1.39)
EDSIS Total Score								
SUCCEAT Workshop	33.32 (15.02)	22.59 (13.24)	20.28 (16.29)	2.421 (0.123)	**57.005 (<0.001)**	0.166 (0.847)	0.75 (0.50; 1.00)	0.83 (0.54; 1.12)
SUCCEAT Online	29.11 (12.70)	19.83 (11.82)	16.60 (11.41)				0.76 (0.42; 1.09)	1.04 (0.60; 1.47)
EDSIS Nutrition ^b^								
SUCCEAT Workshop	16.61 (7.34)	10.09 (5.78)	8.09 (6.28)	1.983 (0.162)	**99.430 (<0.001)**	0.747 (0.475)	0.96 (0.69; 1.22)	1.24 (0.91; 1.56)
SUCCEAT Online	14.38 (6.31)	8.83 (5.83)	7.24 (5.03)				0.91 (0.58; 1.24)	1.24 (0.80; 1.69)
EDSIS Guilt ^b^								
SUCCEAT Workshop	7.80 (4.75)	5.52 (3.96)	5.17 (4.75)	1.547 (0.217)	**19.856 (<0.001)**	0.534 (0.587)	0.52 (0.22; 0.81)	0.55 (0.26; 0.85)
SUCCEAT Online	6.51 (4.23)	5.06 (3.52)	4.33 (3.63)				0.37 (0.08; 0.66)	0.55 (0.19; 0.91)
EDSIS Dysregulated Behavior ^b^								
SUCCEAT Workshop	6.02 (4.96)	4.51 (4.21)	4.53 (4.29)	0.167 (0.683)	**9.651 (<0.001)**	0.197 (0.821)	0.32 (0.12; 0.52)	0.32 (0.05; 0.59)
SUCCEAT Online	5.64 (3.93)	4.50 (3.36)	4.07 (3.74)				0.31 (0.03; 0.59)	0.41 (0.08; 0.74)
EDSIS Social Isolation ^b^								
SUCCEAT Workshop	2.87 (2.60)	2.48 (2.72)	2.49 (3.17)	5.971 (0.016)	**7.190 (0.001)**	2.698 (0.070)	0.14 (−0.16; 0.45)	0.12 (−0,19; 0.45)
SUCCEAT Online	2.64 (2.82)	1.43 (1.95)	0.96 (1.50)				0.50 (0.12; 0.87)	0.73 (0.31; 1.15)
SCL 90-R Total Mean Score								
SUCCEAT Workshop	0.42 (0.36)	0.24 (0.26)	0.33 (0.37)	0.003 (0.953)	**12.864 (<0.001)**	2.261 (0.107)	0.53 (0.32; 0.75)	0.25 (−0.02; 0.54)
SUCCEAT Online	0.43 (0.42)	0.31 (0.36)	0.26 (0.26)				0.29 (0.03; 0.54)	0.45 (0.12; 0.78)
BDI Total Score								
SUCCEAT Workshop	11.60 (7.01)	6.45 (5.43)	6.80 (6.24)	0.155 (0.694)	**25.946 (<0.001)**	2.394 (0.094)	0.80 (0.52; 1.08)	0.72 (0.33; 1.12)
SUCCEAT Online	10.11 (7.03)	7.72 (8.18)	5.74 (5.97)				0.31 (0.04; 0.58)	0.67 (0.30; 1.03)
STAI State Score ^c^								
SUCCEAT Workshop	49.44 (10.14)	39.80 (10.16)	37.85 (10.31)	0.060 (0.807)	**42.972 (<0.001)**	3.312 (0.039)	0.95 (0.57; 1.33)	1.13 (0.73; 1.54)
SUCCEAT Online	45.98 (11.16)	41.84 (12.17)	37.99 (10.31)				0.35 (0.06; 0.65)	0.74 (0.40; 1.08)
STAI Trait Score ^c^								
SUCCEAT Workshop	41.73 (8.61)	37.86 (8.62)	36.16 (9.65)	<0.001 (0.987)	**22.651 (<0.001)**	0.169 (0.845)	0.45 (0.22; 0.68)	0.61 (0.29; 0.92)
SUCCEAT Online	41.25 (10.15)	38.01 (10.57)	36.56 (9.50)				0.31 (0.10; 0.52)	0.48 (0.20; 0.75)

^a^ Primary outcome; ^b^ *p*-values for these subscales are tested against a Bonferroni-adjusted significance level of 0.0125; ^c^ *p*-values for these subscales are tested against a Bonferroni-adjusted significance level of 0.025.

**Table 3:** Means (SDs) and results of the mixed-effects model repeated measures ANOVA for outcomes of the SUCCEAT workshop vs. SUCCEAT online intervention regarding caregiver skills (intention-to-treat analysis).

	Mean (SD)			ANOVA (F, *p*)			Cohen’s d (95% CI Lower; Upper)	
	Baseline (T0)	3M FU (T1)	12M FU (T2)	Group	Time	Time × Group	T0–T1	T0–T2
CASK Total Score								
SUCCEAT Workshop	64.64 (15.34)	75.33 (13.68)	79.53 (15.25)	0.202 (0.654)	**51.351 (<0.001)**	0.636 (0.530)	0.73 (0.40; 1.05)	0.97 (0.56; 1.38)
SUCCEAT Online	65.20 (13.74)	74.08 (12.63)	77.01 (12.87)				0.67 (0.35; 0.99)	0.89 (0.55; 1.22)
CASK Bigger Picture ^a^								
SUCCEAT Workshop	71.01 (17.63)	79.14 (13.68)	83.98 (15.38)	0.530 (0.468)	**23.631 (<0.001)**	1.420 (0.244)	0.51 (0.19; 0.83)	0.78 (0.36; 1.21)
SUCCEAT Online	71.27 (14.22)	78.60 (12.54)	79.28 (13.80)				0.54 (0.21; 0.88)	0.57 (0.24; 0.90)
CASK Selfcare ^a^								
SUCCEAT Workshop	57.23 (18.00)	71.23 (17.00)	76.37 (20.19)	0.222 (0.638)	**59.621 (<0.001)**	0.012 (0.988)	0.80 (0.44; 1.16)	1.00 (0.60; 1.39)
SUCCEAT Online	58.33 (19.05)	72.86 (14.69)	77.57 (15.28)				0.84 (0.53; 1.14)	1.11 (0.70; 1.52)
CASK Biting Tongue ^a^								
SUCCEAT Workshop	53.73 (20.44)	72.20 (17.13)	78.40 (19.11)	0.178 (0.674)	**90.700 (<0.001)**	0.465 (0.629)	0.97 (0.64; 1.30)	1.25 (0.84; 1.64)
SUCCEAT Online	57.03 (22.07)	72.56 (16.78)	78.69 (15.78)				0.78 (0.46; 1.10)	1.10 (0.74; 1.45)
CASK Insight and Acceptance ^a^								
SUCCEAT Workshop	68.67 (20.27)	77.94 (17.99)	82.11 (18.29)	0.005 (0.944)	**32.987 (<0.001)**	<0.001 (1.000)	0.48 (0.23; 0.74)	0.69 (0.39; 1.00)
SUCCEAT Online	68.50 (19.23)	77.76 (14.57)	81.85 (13.21)				0.54 (0.21; 0.86)	0.79 (0.43; 1.16)
CASK Emotional Intelligence ^a^								
SUCCEAT Workshop	66.10 (19.03)	74.54 (16.66)	75.40 (17.07)	2.652 (0.107)	**6.804 (0.001)**	1.606 (0.203)	0.47 (0.15; 0.80)	0.51 (0.13; 0.90)
SUCCEAT Online	65.36 (17.64)	67.59 (18.50)	69.20 (18.59)				0.12 (−0.18; 0.42)	0.21 (−0.10; 0.52)
CASK Frustration Tolerance ^a^								
SUCCEAT Workshop	64.45 (16.40)	73.85 (15.02)	78.13 (15.36)	0.045 (0.833)	**37.927 (<0.001)**	0.347 (0.707)	0.60 (0.26; 0.93)	0.93 (0.51; 1.35)
SUCCEAT Online	64.88 (16.36)	73.90 (14.31)	75.84 (13.87)				0.58 (0.26; 0.91)	0.81 (0.44; 1.19)

^a^*p*-values for these subscales are tested against a Bonferroni-adjusted significance level of 0.008.

## Supplementary Materials

The following are available online at https://www.mdpi.com/2077-0383/9/6/1912/s1, ESM1. Consort checklist and ESM2. Table S1: Means (SDs) and results of the repeated measures ANOVA for outcomes of the SUCCEAT workshop vs. SUCCEAT online intervention regarding caregiving burden and psychopathology (completer analysis), Table S2: Means (SDs) and results of the repeated measures ANOVA for outcomes of the SUCCEAT workshop vs. SUCCEAT online intervention group regarding caregiver skills (completer analysis), Table S3: Results of the mixed-methods ANOVA comparing the SUCCEAT intervention (*n* = 100) and the comparison group (*n* = 49) (intention-to-treat analysis), Table S4: Results of the mixed-methods ANOVA comparing the SUCCEAT intervention and the comparison group (completer analysis),
