# Supplementary material for: Long-Term Efficacy of the Workshop Vs. Online SUCCEAT (Supporting Carers of Children and Adolescents with Eating Disorders) Intervention for Parents: A Quasi-Randomised Feasibility Trial
Source: J Clin Med. 2020 Jun 18;9(6):1912. doi: 10.3390/jcm9061912 (PMC7355675; doi:10.3390/jcm9061912)
Supplement: Supplementary file 1 [file jcm-09-01912-s001.zip › ESM2. Supplementary tables S1-S4.docx]

**Electronic supplementary material**

Supplement to:

Long-term Effectiveness of the Workshop- vs. Online-SUCCEAT (Supporting Carers of Children and Adolescents with Eating Disorders) intervention: A quasi-randomised feasibility trial.

*Journal of Clinical Medicine*

Stefanie Truttmann*, Julia Philipp*, Michael Zeiler, Claudia Franta, Tanja Wittek, Elisabeth Merl, Gabriele Schöfbeck, Doris Koubek, Clarissa Laczkovics, Hartmut Imgart, Annika Zanko, Ellen Auer-Welsbach, Janet Treasure, Andreas F. K. Karwautz and Gudrun Wagner

* contributed equally to this work

Corresponding author:

Gudrun Wagner

Eating Disorders Unit, Department of Child and Adolescent Psychiatry, Medical University of Vienna, Vienna, Austria.

E-Mail: [gudrun.wagner@meduniwien.ac.at](mailto:gudrun.wagner@meduniwien.ac.at)

Contents

Table S1 page 2

Table S2 page 3

Table S3 page 4

Table S4 page 5

**Table S1.** Means (SDs) and results of the repeated measures ANOVA for outcomes of the SUCCEAT workshop vs. SUCCEAT online intervention regarding caregiving burden and psychopathology (completer analysis)

|  | Mean (SD) | | | ANOVA (F, p) | | | Cohens’ dz [95% CI lower; upper] | |
| --- | --- | --- | --- | --- | --- | --- | --- | --- |
|  | Baseline (T0) | 3M FU  (T1) | 12M FU  (T2) | Group | Time | Time x Group | T0-T1 | T0-T2 |
| GHQ Total Score | | | | | | | | |
| SUCCEAT Workshop (n = 38) | 4.40 (3.06) | 1.90 (2.83) | 2.11 (3.24) | 0.411 (.524) | **31.976 (< .001)** | 2.359 (0.098) | 0.76 [0.36; 1.15] | 0.73 [0.28; 1.18] |
| SUCCEAT Online (n = 31) | 4.70 (3.60) | 2.00 (2.99) | 0.72 (1.62) |  |  |  | 0.71 [0.29; 1.33] | 1.37 [0.76; 1.98] |
| EDSIS Total Score | | | | | | | | |
| SUCCEAT Workshop (n = 37) | 34.89 (14.73) | 22.77 (13.56) | 20.49 (17.11) | 2.822 (.098) | **41.080 (< .001)** | 0.433 (.649) | 0.77 [0.48; 1.05] | 0.91 [0.55; 1.26] |
| SUCCEAT Online (n = 30) | 29.17 (14.00) | 19.66 (12.73) | 14.64 (11.64) |  |  |  | 0.82 [0.42; 1.23] | 1.11 [0.54; 1.68] |
| EDSIS Nutrition^a^ | | | | | | |  | |
| SUCCEAT Workshop (n = 38) | 17.04 (7.15) | 10.24 (5.93) | 7.84 (6.22) | 2.874 (.095) | **69.306 (< .001)** | 0.738 (.480) | 0.96 [0.66; 1.26] | 1.36 [0.98; 1.75] |
| SUCCEAT Online (n = 31) | 13.90 (6.53) | 8.57 (6.11) | 6.33 (4.99) |  |  |  | 0.91 (0.51; 1.30] | 1.30 [0.66; 1.94] |
| EDSIS Guilt^a^ | |  |  | | | |  | |
| SUCCEAT Workshop (n = 37) | 8.32 (4.92) | 5.41 (4.04) | 5.14 (4.87) | 1.130 (.292) | **17.013 (< .001)** | 0.551 (.578) | 0.59 [0.25; 0.92] | 0.65 [0.29; 1.01] |
| SUCCEAT Online (n = 30) | 6.83 (4.65) | 5.03 (3.83) | 4.13 (4.03) |  |  |  | 0.46 [0.12; 0.81] | 0.63 [0.18; 1.09] |
| EDSIS Dysregulated Behavior^a^ | | | | | | | |  |
| SUCCEAT Workshop (n = 37) | 6.32 (4.85) | 4.41 (4.08) | 4.62 (4.55) | 0.340 (.562) | **8.234 (< .001)** | 0.650 (.524) | 0.37 [0.15; 0.58] | 0.37 [0.03; 0.71] |
| SUCCEAT Online (n = 30) | 5.73 (4.06) | 4.52 (3.46) | 3.60 (3.91) |  |  |  | 0.39 [0.05; 0.73] | 0.51 [0.06; 0.95] |
| EDSIS Social Isolation^a^ | |  |  |  | | |  | |
| SUCCEAT Workshop (n = 37) | 3.14 (2.70) | 2.70 (2.76) | 2.86 (3.41) | 5.445 (.023) | **5.533 (.005)** | 3.010 (.053) | 0.13 [-0.21; 0.48] | 0.10 [-0.29; 0.48] |
| SUCCEAT Online (n = 30) | 2.80 (2.75) | 1.63 (1.88) | 0.80 (1.37) |  |  |  | 0.69 [0.27; 1.12] | 0.81 [0.31; 1.31] |
| SCL 90-R Total Score | | | | | | | | |
| SUCCEAT Workshop (n = 26) | 0.41 (0.35) | 0.23 (0.22) | 0.35 (0.46) | 0.770 (.385) | **9.924 (< .001)** | **4.467 (.014)** | 0.46 [0.21; 0.71] | 0.21 [-0.20; 0.62] |
| SUCCEAT Online(n = 21) | 0.62 (0.52) | 0.37 (0.41) | 0.24 (0.21) |  |  |  | 0.42 [0.10; 0.74] | 0.83 [0.35; 1.31] |
| BDI Total Score | | | | | | | | |
| SUCCEAT Workshop (n = 26) | 11.63 (8.19) | 6.56 (6.04) | 7.19 (7.37) | 0.017 (.897) | **15.735 (< .001)** | 1.757 (.177) | 0.65 [0.32; 0.98] | 0.53 [0.05; 1.00] |
| SUCCEAT Online (n = 30) | 11.54 (7.39) | 8.19 (9.73) | 4.99 (6.62) |  |  |  | 0.39 [0.07; 0.71] | 0.93 [0.43; 1.43] |
| STAI State Score^b^ | | | | | | | | |
| SUCCEAT Workshop (n = 26) | 50.09 (12.09) | 37.95 (10.34) | 38.41 (10.98) | 0.205 (.652) | **30.482 (< .001)** | 2.619 (.077) | 0.95 [0.43; 1.47] | 0.94 [0.40; 0.94] |
| SUCCEAT Online (n = 30) | 46.74 (10.99) | 40.93 (12.75) | 35.51 (9.60) |  |  |  | 0.51 [0.16; 0.85] | 1.09 [0.57; 1.60] |
| STAI Trait Score^b^ | | | | | | | | |
| SUCCEAT Workshop (n = 26) | 40.43 (8.44) | 36.85 (8.43) | 36.04 (9.99) | 0.002 (.968) | **11.242 (< .001)** | 0.500 (.608) | 0.37 [0.00; 0.73] | 0.42 [0.01; 0.83] |
| SUCCEAT Online (n = 30) | 41.32 (11.35) | 37.50 (11.63) | 34.78 (9.40) |  |  |  | 0.37 [0.11; 0.62] | 0.62 [0.25; 1.00] |

^a^ *p*-values for these subscales are tested against a Bonferroni-adjusted significance level of .0125.

^b^ *p*-values for these subscales are tested against a Bonferroni-adjusted significance level of .025.

**Table S2:** Means (SDs) and results of the repeated measures ANOVA for outcomes of the SUCCEAT workshop vs. SUCCEAT online intervention group regarding caregiver skills (completer analysis)

|  | Mean (SD) | | | ANOVA (F, p) | | | Cohens’ dz [95% CI lower; upper] | | |
| --- | --- | --- | --- | --- | --- | --- | --- | --- | --- |
|  | Baseline (T0) | 3M FU  (T1) | 12M FU  (T2) | Group | Time | Time x Group | T0-T1 | | T0-T2 |
| CASK Total Score | | | | | | | | | |
| SUCCEAT Workshop (n = 37) | 65.23 (15.67) | 75.52 (13.56) | 78.33 (16.61) | 0.006 (.940) | **26.591 (< .001)** | 0.190 (.828) | 0.73 [0.36; 1.10] | | 0.81 [0.35; 1.28] |
| SUCCEAT Online (n = 31) | 65.94 (13.65) | 74.07 (13.41) | 78.41 (13.23) |  |  |  | 0.66 [0.29; 1.03] | | 0.93 [0.46; 1.40] |
| CASK Bigger Picture^a^ | | | | | | | | | |
| SUCCEAT Workshop (n = 37) | 72.54 (17.64) | 79.90 (12.91) | 82.66 (16.86) | 0.239 (.627) | **11.719 (< .001)** | 0.104 (.901) | 0.52 [0.14; 0.89] | | 0.59 [0.13; 1.05] |
| SUCCEAT Online (n = 31) | 72.12 (12.39) | 78.44 (13.19) | 80.45 (14.56) |  |  |  | 0.51 [0.17; 0.86] | | 0.61 [0.16; 1.07] |
| CASK Selfcare^a^ | | | | | | | |  | |
| SUCCEAT Workshop (n = 38) | 56.02 (18.31) | 71.01 (17.59) | 75.33 (22.13) | 0.405 (.526) | **38.925 (< .001)** | 0.458 (.633) | 0.80 [0.40; 1.21] | | 0.95 [0.49; 1.40] |
| SUCCEAT Online (n = 31) | 57.93 (20.50) | 71.21 (16.61) | 80.08 (16.13) |  |  |  | 0.81 [0.45; 1.17] | | 1.19 [0.65; 1.74] |
| CASK Biting Tongue^a^ | | | | | | | | | |
| SUCCEAT Workshop (n = 37) | 52.34 (20.70) | 73.06 (17.27) | 76.85 (21.04) | 0.051 (.822) | **48.532 (<. 001)** | 0.783 (.459) | 1.07 [0.70; 1.45] | | 1.17 [0.70; 1.65] |
| SUCCEAT Online (n = 29) | 56.21 (21.00) | 70.80 (17.29) | 77.82 (16.26) |  |  |  | 0.81 [0.40; 1.22] | | 1.11 [0.59; 1.64] |
| CASK Insight and Acceptance^a^ | | | | | | | | | |
| SUCCEAT Workshop (n = 38) | 69.56 (20.57) | 79.21 (17.59) | 80.75 (20.10) | 0.024 (.878) | **19.234 (< .001)** | 0.504 (.605) | 0.49 [0.21; 0.78] | | 0.55 [0.22; 0.88] |
| SUCCEAT Online (n = 31) | 68.23 (20.53) | 76.99 (15.74) | 82.58 (14.08) |  |  |  | 0.59 [0.21; 0.96] | | 0.80 [0.33; 1.28] |
| CASK Emotional Intelligence^a^ | | | | | | | | | |
| SUCCEAT Workshop (n = 38) | 67.13 (19.66) | 74.29 (17.59) | 74.63 (18.34) | 0.649 (.423) | 2.736 (.068) | 0.615 (.542) | 0.40 [0.06; 0.74] | | 0.39 [-0.05; 0.83] |
| SUCCEAT Online (n = 31) | 67.53 (17.05) | 69.67 (16.02) | 70.63 (20.87) |  |  |  | 0.10 [-0.24; 0.45] | | 0.16 [-0.26; 0.58] |
| CASK Frustration Tolerance^a^ | | | | | | | | | |
| SUCCEAT Workshop (n = 38) | 65.22 (16.85) | 74.82 (14.05) | 79.00 (16.15) | 0.004 (.953) | **23.904 (< .001)** | 0.041 (.960) | 0.64 (0.27; 1.01] | | 0.83 [0.34; 1.32] |
| SUCCEAT Online (n = 31) | 65.58 (15.41) | 74.04 (14.88) | 78.90 (13.41) |  |  |  | 0.60 [0.23; 0.96] | | 0.92 [0.42; 1.42] |

^a^ *p*-values for these subscales are tested against a Bonferroni-adjusted significance level of .008.

**Table S3.** Results of the mixed-methods ANOVA comparing the SUCCEAT intervention (n = 100) and the comparison group (n = 49) (intention-to-treat analysis)

|  | Mean (SD) | | | ANOVA (F, p) | | | Cohens’ dz [95% CI lower; upper] | |
| --- | --- | --- | --- | --- | --- | --- | --- | --- |
|  | Baseline (T0) | 3M FU  (T1) | 12M FU  (T2) | Group | Time | Time x Group | T0-T1 | T0-T2 |
| GHQ Total Score | | | | | | | | |
| SUCCEAT Groups | 4.37 (3.45) | 1.99 (2.82) | 1.67 (2.58) | **8.242 (.005)** | **51.249 (< .001)** | 0.850 (.429) | 0.75 [0.50; 1.00] | 0.88 [0.59; 1.18] |
| Comparison Group | 5.65 (4.06) | 3.56 (3.49) | 2.45 (2.82) |  |  |  | 0.55 [0.26; 0.84] | 0.90 [0.52; 1.27] |
| EDSIS Total Score | | | | | | | | |
| SUCCEAT Groups | 31.22 (14.00) | 21.21 (12.58) | 18.44 (14.11) | **4.882 (.029)** | **75.886 (< .001)** | 0.069 (.933) | 0.75 [0.55; 0.95] | 0.91 [0.67; 1.15] |
| Comparison Group | 35.46 (14.91) | 25.99 (14.70) | 23.44 (15.08) |  |  |  | 0.64 [0.42; 0.86] | 0.80 [0.50; 1.10] |
| SCL 90-R Total Mean Score | | | | | | | | |
| SUCCEAT Groups | 0.42 (0.39) | 0.28 (0.31) | 0.30 (0.32) | **8.288 (.005)** | **10.295 (< .001)** | 1.252 (.287) | 0.41 [0.23; 0.58] | 0.36 [0.14; 0.57] |
| Comparison Group | 0.53 (0.37) | 0.47 (0.38) | 0.45 (0.34) |  |  |  | 0.17 [-0.06; 0.39] | 0.23 [-0.03; 0.48] |
| BDI Total Score | | | | | | | | |
| SUCCEAT Groups | 10.86 (7.02) | 7.08 (6.93) | 6.27 (6.10) | **9.284 (.003)** | **36.686 (< .001)** | 1.363 (.258) | 0.54 [0.34; 0.74] | 0.70 [0.43; 0.96] |
| Comparison Group | 13.83 (7.99) | 11.05 (7.43) | 8.31 (5.70) |  |  |  | 0.36 [0.10; 0.62] | 0.77 [0.46; 1.09] |
| STAI State Score^a^ | | | | | | | | |
| SUCCEAT Groups | 47.71 (10.75) | 40.82 (11.20) | 37.92 (10.26) | **10.286 (.002)** | **44.446 (< .001)** | 1.890 (.153) | 0.63 [0.39; 0.86] | 0.93 [0.67; 1.19] |
| Comparison Group | 50.85 (11.98) | 47.34 (12.12) | 43.64 (10.03) |  |  |  | 0.29 [0.06; 0.52] | 0.65 [0.36; 0.93] |
| STAI Trait Score^a^ | | | | | | | | |
| SUCCEAT Groups | 41.49 (9.37) | 37.94 (9.60) | 36.36 (9.53) | **12.297 (.001)** | **24.701 (< .001)** | 0.821 (.441) | 0.37 [0.22; 0.53] | 0.54 [0.34; 0.75] |
| Comparison Group | 46.15 (10.68) | 43.33 (10.89) | 42.64 (10.11) |  |  |  | 0.26 [0.08; 0.44] | 0.34 [0.13; 0.54] |
| CASK Total Score | | | | | | | | |
| SUCCEAT Groups | 64.92 (14.49) | 74.65 (13.11) | 78.27 (14.09) | 3.720 (.056) | **45.010 (< .001)** | **5.570 (.004)** | 0.70 [0.47; 0.93] | 0.93 [0.67; 1.20] |
| Comparison Group | 64.85 (12.46) | 70.75 (11.52) | 70.96 (11.79) |  |  |  | 0.49 [0.24; 0.74] | 0.50 [0.21; 0.79] |

^a^ *p*-values for these subscales are tested against a Bonferroni-adjusted significance level of .025.

**Table S4.** Results of the mixed-methods ANOVA comparing the SUCCEAT intervention and the comparison group (completer analysis)

|  | Mean (SD) | | | ANOVA (F, p) | | | Cohens’ dz [95% CI lower; upper] | |
| --- | --- | --- | --- | --- | --- | --- | --- | --- |
|  | Baseline (T0) | 3M FU  (T1) | 12M FU  (T2) | Group | Time | Time x Group | T0-T1 | T0-T2 |
| GHQ Total Score | | | | | | | | |
| SUCCEAT Groups (n = 69) | 4.53 (3.29) | 1.93 (3.71) | 1.48 (2.71) | 2.338 (.130) | **28.622 (<. 001)** | 1.796 (.169) | 0.74 [0.45; 1.03] | 1.01 [0.64; 1.38] |
| Comparison Group (n = 21) | 5.29 (3.62) | 3.71 (4.21) | 1.57 (2.52) |  |  |  | 0.54 [0.15; 0.93] | 0.79 [0.24; 1.34] |
| EDSIS Total Score | | | | | | | | |
| SUCCEAT Groups (n = 67) | 32.33 (14.58) | 21.38 (13.19) | 17.87 (15.09) | 2.262 (.136) | **36.415 (< .001)** | 0.158 (.854) | 0.78 [0.55; 1.02] | 0.98 [0.68; 1.27] |
| Comparison Group (n = 20) | 36.09 (10.41) | 25.75 (15.18) | 19.16 (15.09) |  |  |  | 0.65 [0.36; 0.93] | 0.71 [0.21; 1.21] |
| SCL 90-R Total Mean Score | | | | | | | | |
| SUCCEAT Groups (n = 47) | 0.51 (0.45) | 0.29 (0.32) | 0.30 (0.37) | 1.698 (.197) | **3.241 (.042)** | 2.615 (.077) | 0.44 [0.24; 0.64] | 0.53 [0.20; 0.86] |
| Comparison Group (n = 21) | 0.49 (0.37) | 0.48 (0.49) | 0.47 (0.42) |  |  |  | 0.07 [-0.23; 0.38] | 0.00 [-0.37; 0.36] |
| BDI Total Score | | | | | | | | |
| SUCCEAT Groups (n = 56) | 11.58 (7.70) | 7.43 (8.19) | 6.01 (7.00) | 0.713 (.401) | **17.007 (< .001)** | 0.102 (.903) | 0.51 [0.27; 0.75] | 0.72 [0.38; 1.06] |
| Comparison Group (n = 17) | 13.24 (7.73) | 9.19 (7.17) | 6.91 (6.28) |  |  |  | 0.60 [0.15; 1.04] | 0.66 [0.16; 1.16] |
| STAI State Score^a^ | | | | | | | | |
| SUCCEAT Groups (n = 56) | 48.30 (11.53) | 39.55 (11.69) | 36.86 (10.27) | 1.121 (.293) | **21.837 (< .001)** | 0.506 (.604) | 0.70 [0.40; 1.00] | 1.00 [0.64; 1.37] |
| Comparison Group (n = 18) | 49.22 (12.00) | 42.85 (14.87) | 40.78 (11.35) |  |  |  | 0.50 [0.17; 0.83] | 0.52 [0.06; 0.99] |
| STAI Trait Score^a^ | | | | | | | | |
| SUCCEAT Groups (n = 56) | 40.91 (10.03) | 37.20 (10.18) | 35.36 (9.61) | 3.352 (.071) | **10.060 (< .001)** | 0.153 (.858) | 0.37 [0.16; 0.58] | 0.53 [0.25; 0.81] |
| Comparison Group (n = 18) | 45.28 (11.31) | 41.44 (14.03) | 40.78 (12.59) |  |  |  | 0.31 [0.04; 0.58] | 0.27 [-0.05; 0.59] |
| CASK Total Score | | | | | | | | |
| SUCCEAT Groups (n = 68) | 65.55 (14.68) | 74.86 (13.41) | 78.37 (15.05) | 1.656 (.202) | **16.218 (< .001)** | 2.505 (.085) | 0.70 [0.44; 0.96) | 0.86 [0.53; 1.19] |
| Comparison Group (n = 22) | 65.64 (12.03) | 71.78 (10.65) | 70.84 (11.16) |  |  |  | 0.45 [0.09; 0.80] | 0.30 [-0.09; 0.70] |

^a^ *p*-values for these subscales are tested against a Bonferroni-adjusted significance level of .025.
