# Supplementary material for: Long-Term Efficacy of the Workshop Vs. Online SUCCEAT (Supporting Carers of Children and Adolescents with Eating Disorders) Intervention for Parents: A Quasi-Randomised Feasibility Trial
Source: J Clin Med. 2020 Jun 18;9(6):1912. doi: 10.3390/jcm9061912 (PMC7355675; doi:10.3390/jcm9061912)
Supplement: Supplementary file 1 [file jcm-09-01912-s001.zip › Supplementary - consort checklist final - Copy.doc]

**CONSORT 2010 checklist of information to include when reporting a pilot or feasibility randomized trial in a journal or conference abstract**

| **Item** | **Description** | **Reported on line number** |
| --- | --- | --- |
| Title | Identification of study as randomised pilot or feasibility trial | page 1 |
| Authors * | Contact details for the corresponding author | page 1 |
| Trial design | Description of pilot trial design (eg, parallel, cluster) | page 3 |
| Methods |  |  |
| Participants | Eligibility criteria for participants and the settings where the pilot trial was conducted | page 3 & 4 |
| Interventions | Interventions intended for each group | page 4 |
| Objective | Specific objectives of the pilot trial | page 2 & 3 |
| Outcome | Prespecified assessment or measurement to address the pilot trial objectives** | Page 4 & 5 |
| Randomization | How participants were allocated to interventions | page 3 |
| Blinding (masking) | Whether or not participants, care givers, and those assessing the outcomes were blinded to group assignment | page 3 |
| Results |  | Page 6 |
| Numbers randomized | Number of participants screened and randomised to each group for the pilot trial objectives** | page 6 & 7  figure 1 |
| Recruitment | Trial status† | n.a. |
| Numbers analysed | Number of participants analysed in each group for the pilot objectives** | page 6 & 7  figure 1 |
| Outcome | Results for the pilot objectives, including any expressions of uncertainty** | page 8 - 11  table 1,2 & 3 |
| Harms | Important adverse events or side effects | page 12 |
| Conclusions | General interpretation of the results of pilot trial and their implications for the future definitive trial | page 14 & 15 |
| Trial registration | Registration number for pilot trial and name of trial register | page 3 |
| Funding | Source of funding for pilot trial | page 16 |

Citation: Eldridge SM, Chan CL, Campbell MJ, Bond CM, Hopewell S, Thabane L, et al. CONSORT 2010 statement: extension to randomised pilot and feasibility trials. BMJ. 2016;355.

**this item is specific to conference abstracts*

***Space permitting, list all pilot trial objectives and give the results for each. Otherwise, report those that are a priori agreed as the most important to the decision to proceed with the future*

*definitive RCT.*

*†For conference abstracts.*
